# Mosquito‐Borne Viruses of Clinical Significance

**DOI:** 10.1002/hsr2.71814

**Published:** 2026-02-11

**Authors:** Donath Damian

**Affiliations:** ^1^ Department of Biochemistry, Mbeya College of Health and Allied Sciences University of Dar es Salaam Mbeya Tanzania

**Keywords:** clinical impact, diagnostic challenges, geographic distribution, mosquito‐borne viruses, research gaps, treatment options

## Abstract

**Background:**

Mosquito‐borne viruses represent a significant public health concern globally, with rising incidence rates leading to increased morbidity and mortality. This review aims to synthesize current knowledge regarding mosquito‐borne viruses, focusing on their geographic distribution, clinical impact, diagnostic challenges, treatment options, and existing research gaps.

**Methods:**

A comprehensive literature search was conducted across multiple electronic databases, including PubMed, Web of Science, Scopus, Google Scholar, and the Cochrane Library. Studies published from January 2000 to the present were included, focusing on peer‐reviewed articles related to mosquito‐borne viruses. Data extraction and synthesis followed a structured methodology, assessing both the quality of included studies and the themes relevant to mosquito‐borne viruses.

**Results:**

The review revealed significant geographic expansion of mosquito‐borne viruses, facilitated by climate change and urbanization, affecting regions previously considered low risk. Clinical manifestations varied widely, with vulnerable populations, such as children and the elderly, being at higher risk for severe outcomes. Diagnostic methods, while improving, faced limitations in sensitivity and specificity. Treatment primarily focused on symptomatic relief, with limited antiviral options currently available. Notable gaps in research were identified, particularly in the development of effective vaccines and rapid diagnostic tools.

**Conclusions:**

The findings underscore the urgent need for integrated public health strategies to combat mosquito‐borne viruses. Enhancing surveillance systems, improving diagnostic capabilities, expanding treatment options, and fostering vaccine development are critical steps. Addressing the identified research gaps will help mitigate the impacts of mosquito‐borne viruses on global health.

## 1 Introduction

Mosquito‐borne viruses (MBVs) represent a significant threat to global public health, with several pathogens causing substantial morbidity and mortality. This review focuses specifically on major MBVs of global significance, including dengue virus, Zika virus, yellow fever virus, chikungunya virus, and West Nile virus. Each of these viruses exhibits unique epidemiological characteristics and clinical manifestations. For example, over 200,000 cases of yellow fever were reported in 2024 alone, highlighting the ongoing threat of these viruses to public health. Chikungunya, caused by the Chikungunya virus and transmitted by Aedes mosquitoes, is one of the most common mosquito‐borne diseases globally, with high numbers of cases and deaths. As of December 2024, there have been approximately 480,000 cases and over 200 deaths=. Dengue fever, transmitted by Aedes mosquitoes, has seen a dramatic increase in incidence over recent decades, with estimates suggesting 390 million infections annually [1]. Similarly, Zika virus, also spread by Aedes mosquitoes, garnered global attention during the 2015 outbreak in Brazil, where it was linked to severe birth defects, underscoring its potential for devastating consequences [2]. West Nile virus, primarily transmitted by Culex mosquitoes, remains endemic in many regions, particularly in North America, leading to thousands of cases each year [3].

The rising incidence of MBVs can be attributed to various factors, including climate change, urbanization, and increased global travel, which facilitate the spread of both vectors and viruses [4]. As urban areas expand into mosquito habitats, populations become increasingly exposed to these pathogens. Climate change, including global warming, plays a crucial role in altering mosquito and pathogen dynamics, affecting vector habitats, mosquito breeding patterns, and pathogen survival rates. For example, increased temperatures can extend the range and transmission season of mosquitoes, facilitating the spread of diseases like dengue, Zika, and Chikungunya [5]. The World Health Organization [6] has identified MBVs as critical public health threats, emphasizing the need for enhanced surveillance and response strategies to mitigate outbreaks [7]. Moreover, the economic burden of MBV‐related diseases places significant pressure on healthcare systems, especially in low‐ and middle‐income countries, where resources are limited, and healthcare access is often inadequate [8].

This review aims to synthesize current knowledge regarding major MBVs, specifically dengue, Zika, chikungunya, yellow fever, and West Nile virus, focusing on their geographic distribution, clinical impact, diagnostic challenges, and treatment options. By providing a comprehensive overview, we aim to identify critical research gaps, particularly in the development of improved diagnostic tools and effective vaccines. Addressing these gaps is essential for advancing the field and enhancing public health responses to the ongoing threat posed by MBVs.

## 2 Materials and Methods

### 2.1 Research Questions

To achieve this objective, the review addressed several key research questions: What is the geographic distribution of the major MBVs included in this review (dengue, Zika, chikungunya, yellow fever, and West Nile virus)? What were the clinical impacts and manifestations associated with these viruses? What diagnostic tools were available, and what limitations did they face? Additionally, the review explored existing treatment options for MBV infections and ongoing research in this area, culminating in a discussion of significant research gaps that warranted further investigation.

### 2.2 Search Strategy

A systematic literature search was conducted across multiple electronic databases, including PubMed, Web of Science, Scopus, Google Scholar, and the Cochrane Library. The search strategy utilized a combination of keywords and Medical Subject Headings (MeSH) terms pertinent to MBVs, such as “MBVs,” “dengue virus,” “Zika virus,” “West Nile virus,” “Chikungunya virus,” and “yellow fever,” along with terms such as “geographic distribution,” “clinical impact,” “diagnostic methods,” and “treatment options.” The search was limited to articles published in English from January 2000 to the present, ensuring a focus on recent and relevant research.

Studies were selected based on their relevance to understanding the epidemiology, clinical management, and treatment advancements for the five major MBVs of focus, as well as their geographic distribution and diagnostic methodologies. This approach ensured that the search encompassed both global and region‐specific insights into MBVs, their impact on public health, and the current state of scientific knowledge.

The search strategy aimed to gather comprehensive and up‐to‐date information on these selected MBVs, ensuring that the resulting studies reflected the most current developments in the field.

### 2.3 Inclusion and Exclusion Criteria

Studies were included if they met the following criteria: (1) published in English; (2) peer‐reviewed; (3) focused on one or more of the five target viruses (dengue, Zika, chikungunya, yellow fever, and West Nile); and (4) addressed at least one of the following areas geographic distribution, clinical manifestations, diagnostics, treatment options, or research gaps. Eligible study designs included original research, systematic or narrative reviews, meta‐analyses, and case studies. Conversely, studies were excluded if they (1) focused solely on non‐MBVs or other vector‐borne diseases, (2) lacked sufficient data or methodological detail, or (3) were not accessible in full‐text format.

### 2.4 Study Selection

The study selection process followed a structured approach. Initially, titles and abstracts of identified articles were screened for relevance. Full‐text articles were then assessed to confirm eligibility based on the predetermined criteria. Following this, data extraction was conducted on eligible studies, capturing essential details, such as author(s), year of publication, study design, key findings, and their relevance to the research questions posed.

### 2.5 Data Synthesis

Data synthesis involved a narrative approach, summarizing findings from the included studies. This synthesis focused on elucidating geographic distribution patterns of the five major MBVs, clinical manifestations and epidemiological trends, the diagnostic tools available along with their limitations, treatment options, and ongoing research efforts. Additionally, the synthesis identified critical research gaps that needed to be addressed to advance the field.

### 2.6 Quality Assessment

To ensure the robustness of the review, the methodological quality of the included studies was assessed using established tools. The Newcastle–Ottawa Scale (NOS) was used for evaluating observational studies, while the Cochrane Risk of Bias Tool was applied to randomized controlled trials. Review articles and meta‐analyses were assessed for methodological rigor using adapted AMSTAR criteria. This quality assessment categorized studies as high, moderate, or low quality based on their methodological rigor, providing context for the findings.

## 3 Results

### 3.1 Geographic Distribution of Mosquito‐Borne Viruses of Clinical Significance

#### 3.1.1 Global Overview

**FIGURE 1:**
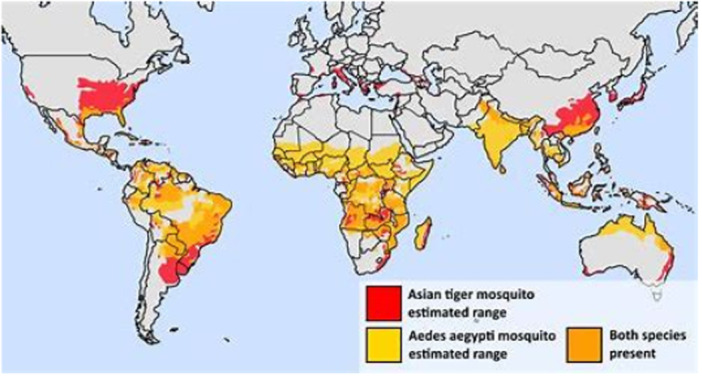
Map Illustrating the geographic distribution of mosquito vectors for mosquito‐borne viruses (MBVs).

MBVs are distributed across various regions of the world, with their prevalence heavily influenced by environmental factors and human activities (Figure 1). Major MBVs, including dengue, Zika, West Nile, Chikungunya, and yellow fever, demonstrate distinct geographic ranges.

Dengue virus is predominantly found in tropical and subtropical regions, with high incidence rates reported in Southeast Asia, the Western Pacific, the Americas, and parts of Africa [1]. It affects more than 100 countries and causes an estimated 390 million infections annually, with 96 million manifesting clinically [1]. Zika virus, initially identified in Uganda and Tanzania, has since spread to many countries in the Americas, particularly during the 2015 outbreak, which saw extensive transmission in Brazil and beyond [2]. In Brazil alone, over 1.5 million suspected cases were recorded during the 2015–2016 epidemic [2]. West Nile virus, on the other hand, is endemic to North America, Europe, and parts of Asia, with a marked presence in the United States, particularly in the Midwest and Southern states [3]. In 2020, the U.S. reported over 1000 confirmed cases and 47 deaths from West Nile virus [3].

Chikungunya virus, primarily transmitted by Aedes mosquitoes, has expanded its reach globally since its initial emergence in Africa and Asia. The virus was first reported in the Americas in 2013, and large‐scale outbreaks occurred in the Caribbean, Latin America, and parts of the United States [3]. Chikungunya remains endemic in many tropical regions, but its seasonal transmission has now been observed in subtropical areas, emphasizing the shift in geographic distribution patterns. Major outbreaks have affected millions globally, particularly in the Americas [3].

The yellow fever virus is endemic in sub‐Saharan Africa, parts of South America, and Central America, with sporadic outbreaks reported globally. In 2024, over 200,000 cases of yellow fever were reported, demonstrating the persistence and ongoing risks of the virus in these regions [9]. Despite the existence of a vaccine, challenges in distribution and surveillance have contributed to recent outbreaks in more than 30 countries [9]. In recent years, the introduction of yellow fever vaccines has been a crucial strategy in controlling outbreaks, but challenges in vaccine distribution and surveillance persist in certain endemic areas [10].

The global distribution of these viruses can be visually represented on a map, highlighting regions of endemicity and areas affected by recent outbreaks, underscoring how factors such as climate change, urbanization, and human movement have influenced the shifting geographic ranges of these MBVs.

#### 3.1.2 Climate and Environmental Factors

**FIGURE 2:**
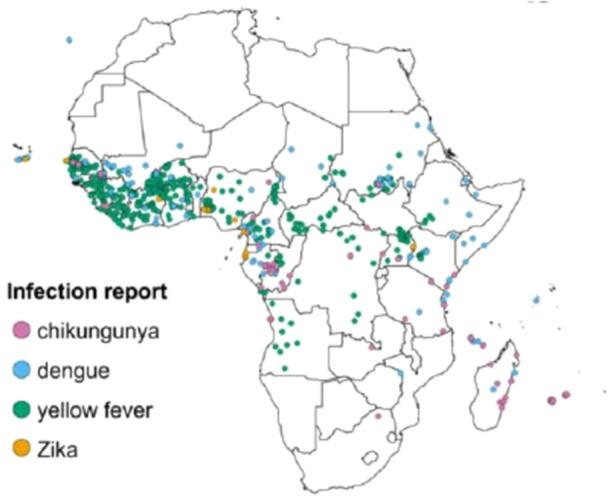
Map Illustrating the geographic distribution of mosquito‐borne viruses (MBVs) in Africa.

Climate change and environmental factors significantly influence the distribution of MBVs (Figure 2). Rising temperatures can expand the habitats suitable for mosquito vectors, enabling them to thrive in regions previously too cold for survival. For example, studies have shown that *Aedes aegypti*, the primary vector for dengue, Zika, and yellow fever viruses, can establish populations in higher altitudes and latitudes due to warmer temperatures [4]. The expansion of *Aedes aegypti* has been documented in regions above 1500 m in elevation, particularly in parts of South America and East Africa [4]. Similarly, *Aedes albopictus*, the vector for Chikungunya virus, has expanded its range in response to changing climate conditions, contributing to outbreaks in areas previously unaffected by the disease.

Moreover, changes in rainfall patterns can affect mosquito breeding sites. Excessive rainfall can create more standing water for mosquito breeding, leading to increased mosquito populations and a higher likelihood of disease transmission. Conversely, drought can disrupt water management systems and increase the risk of mosquito proliferation in urban areas, where stagnant water accumulates in containers, discarded tires, or poorly maintained infrastructure [8]. This is particularly relevant for yellow fever and Chikungunya, which are transmitted by the same mosquito species that thrive in urban environments with poor sanitation. For instance, increased rainfall has been linked to dengue outbreaks in over 15 tropical countries during La Niña years [8].

Additionally, urbanization and land use changes, often exacerbated by climate change, can lead to increased human‐mosquito interactions, further facilitating the transmission of MBVs. As urban areas expand and encroach on natural habitats, there are more opportunities for mosquitoes to interact with humans, particularly in areas with poor sanitation or water management. For example, yellow fever outbreaks have been linked to rapid urbanization in parts of Africa and South America, where mosquito populations are more concentrated due to human activities [9]. Studies have shown that population density increases of 20% or more are associated with a rise in Chikungunya transmission in urban centers [9].

The intersection of these environmental factors underscores the need for integrated approaches in monitoring and managing the risks associated with mosquito‐borne diseases. Effective strategies must account for climate projections, urban planning, and vector control efforts to mitigate the growing threat of MBVs worldwide, particularly for dengue, Zika, Chikungunya, and yellow fever.

#### 3.1.3 Clinical Impact of Mosquito‐Borne Viruses of Clinical Significance

##### 3.1.3.1 Epidemiology

MBVs pose significant public health challenges globally, with their incidence and prevalence rates varying by virus and geographic region. Dengue virus is one of the most widespread MBVs, causing an estimated 390 million infections annually, with approximately 96 million cases manifesting clinically [1]. The incidence of dengue has surged dramatically over the past few decades, with the World Health Organization [6] reporting an eight‐fold increase in cases since 2000, affecting over 100 countries worldwide [7]. Zika virus, while historically less prevalent, gained notoriety during the 2015–2016 epidemic, particularly in the Americas, where over 1.5 million suspected cases were reported in Brazil alone [2]. West Nile virus has been endemic in the United States since its introduction in 1999, with thousands of cases reported each year. For instance, in 2020, over 1000 cases were confirmed, resulting in 47 deaths [3]. In addition, Chikungunya virus, initially restricted to Africa and Asia, has seen global expansion in recent years, with major outbreaks in the Americas, Caribbean, and Europe [3]. Similarly, Yellow fever, once thought to be controlled, still causes significant outbreaks, with over 200,000 cases reported in 2024 alone [9]. These epidemiological trends highlight the increasing burden of MBVs on healthcare systems and the urgent need for effective public health interventions.

##### 3.1.3.2 Clinical Manifestations

The clinical manifestations of MBVs vary significantly depending on the specific virus and the host's immune response. Dengue virus typically causes a range of symptoms, including high fever, severe headache, retro‐orbital pain, joint and muscle pain, rash, and mild bleeding, such as nosebleeds and gum bleeding. In severe cases, dengue can result in life‐threatening complications, such as plasma leakage, which can lead to dengue hemorrhagic fever and dengue shock syndrome. These severe forms require immediate medical intervention to prevent death [7]. Zika virus, by contrast, is often asymptomatic, but when symptoms occur, they generally include mild fever, rash, conjunctivitis, muscle and joint pain, and headache. Although severe complications are rare, Guillain‐Barré syndrome and birth defects like congenital Zika syndrome, including microcephaly, have been linked to maternal infection during pregnancy, raising concerns about its impact on infant health [2]. West Nile virus affects approximately 80% of infected individuals asymptomatically, but symptomatic cases can lead to West Nile fever, characterized by fever, headache, body aches, and skin rash. In more severe cases, the infection can progress to neuroinvasive disease, such as meningitis or encephalitis, resulting in long‐term neurological complications or death [3].

Chikungunya virus typically causes high fever, severe joint pain (often affecting the hands and feet), rash, and muscle pain. The joint pain can be prolonged, often lasting for weeks to months in some individuals, which significantly impacts the quality of life and causes long‐term morbidity [3]. While not usually fatal, the persistent joint pain makes Chikungunya a considerable cause of disability in affected populations. Finally, yellow fever symptoms begin with fever, chills, headache, back pain, and muscle aches, but in severe cases, it can progress to jaundice, bleeding, and organ failure, which may lead to death. Yellow fever has a high mortality rate in its severe form, particularly in regions with inadequate healthcare infrastructure and low vaccination coverage, where the case fatality rate can reach 50% [9]. These diverse clinical manifestations underscore the serious health impacts of MBVs, particularly in regions with inadequate healthcare systems.

##### 3.1.3.3 At‐Risk Populations

Certain populations are at heightened risk for severe outcomes from MBV infections. Children, particularly those who have previously been infected with dengue, are especially vulnerable to severe forms of the disease due to the phenomenon of antibody‐dependent enhancement, which increases the risk of complications [7]. The elderly, as well as vulnerable populations, such as children and the elderly, being at higher risk, face increased risks, as aging often correlates with a weakened immune system and the presence of comorbidities that can exacerbate the severity of infections, particularly for dengue and West Nile viruses [4]. Additionally, immunocompromised individuals, such as those undergoing cancer treatments, organ transplant recipients, or people living with HIV/AIDS, are at a greater risk for severe manifestations of MBV infections. These individuals may experience prolonged illness and a higher likelihood of complications, highlighting the need for targeted public health strategies to protect vulnerable groups [8].

#### 3.1.4 Diagnostic Challenges for Mosquito‐Borne Viruses (MBVs)

##### 3.1.4.1 Current Diagnostic Methods and Challenges

The diagnosis of MBVs is essential for effective treatment and public health responses. Diagnostic methods include serological assays and polymerase chain reaction (PCR), both of which have their unique benefits, limitations, and accessibility issues depending on the region and virus in question.

Serological tests, especially enzyme‐linked immunosorbent assays (ELISA), are commonly used for detecting antibodies, such as IgM and IgG, that indicate recent or past infections [7]. These tests are particularly useful for diagnosing dengue, Zika, Chikungunya, and Yellow Fever. For dengue and Zika, IgM antibodies typically appear during the early stages of infection, while IgG antibodies are associated with past exposure. However, serological tests can be less effective when cross‐reactivity occurs, particularly between dengue and Zika, or Yellow Fever and Chikungunya viruses [4]. This is a significant challenge for accurate diagnosis, especially in regions where multiple MBVs co‐circulate. Moreover, the effectiveness of these tests can vary depending on the stage of infection, with a higher likelihood of detecting antibodies after a few days of symptoms [4]. Availability of serological tests is often more widespread in developed countries, but can be limited in low‐resource settings, where diagnostic infrastructure is inadequate. In these areas, the lack of access to laboratory facilities can hinder timely diagnosis and response.

PCR is a more direct diagnostic method that detects viral genetic material in clinical samples like blood, urine, and cerebrospinal fluid. PCR is highly effective during the acute phase of infection when viral loads are high and before antibodies are produced [8]. It is particularly useful for diagnosing Zika, West Nile, and Chikungunya viruses. PCR can also be used to confirm Yellow Fever during the early stages of infection. However, PCR is often resource‐intensive, requiring specialized equipment and trained technicians, which makes it less accessible in rural or low‐income regions [8]. Cost is another factor that limits PCR use in resource‐limited areas. Despite these challenges, PCR is a critical tool in regions with advanced healthcare systems, as it provides early and precise identification of viruses, enabling timely intervention.

In contrast, rapid diagnostic tests (RDTs) offer a quicker, more affordable alternative to PCR and serological assays. These tests are especially valuable in areas where laboratory infrastructure is lacking. RDTs for Chikungunya, Yellow Fever, and dengue have been developed and are used in field settings, providing results within minutes. However, the accuracy of RDTs can vary, with some tests having lower sensitivity and specificity than PCR or ELISA, particularly for Zika or West Nile infections, where the risk of false negatives or false positives can be higher [2]. Despite these limitations, RDTs offer a practical solution in regions with limited access to laboratory testing, though there are concerns regarding their reliability during outbreaks of multiple MBVs.

A major challenge in diagnosing Yellow Fever and Chikungunya lies in the timing of the test. In the early stages of Yellow Fever, PCR can be highly effective in detecting the virus in blood samples [9]. However, once symptoms progress to jaundice and organ failure, diagnostic tests can become less reliable, and clinical judgment is critical in managing severe cases. Chikungunya, known for causing debilitating joint pain, can often be misdiagnosed as other diseases with similar symptoms, like dengue or Zika. Therefore, accurate diagnosis relies heavily on differentiating between these viruses based on clinical presentation and laboratory tests.

##### 3.1.4.2 Effectiveness and Availability of Diagnostic Methods

While serological tests and PCR remain the gold standard for diagnosing MBVs, the availability and effectiveness of these methods depend on the region, healthcare infrastructure, and the virus in question [2]. In high‐resource settings, such as the United States and Europe, diagnostic methods are widely available and highly effective, enabling rapid responses to outbreaks. In low‐ and middle‐income countries, however, limited access to diagnostic technologies remains a major barrier to early detection and management. For example, countries in sub‐Saharan Africa and Southeast Asia often lack the laboratory capacity for PCR, and the availability of ELISA tests can be sporadic, depending on funding and infrastructure [8]. The emergence of RDTs has improved accessibility in these regions, but concerns about their accuracy persist.

In regions where Yellow Fever and Chikungunya remain endemic, vaccination programs and vector control efforts are critical, as these diseases are often most effectively controlled by preventative measures rather than through diagnostic interventions alone [9]. Therefore, improving both the availability of diagnostic tests and preventive measures (like vaccines and vector control) remains crucial to mitigating the public health burden of MBVs.

##### 3.1.4.3 Need for Improvement

The need for improved diagnostic tools for MBVs is critical, particularly for ensuring timely and accurate diagnosis in clinical settings. RDTs have emerged as a promising solution, offering the potential for point‐of‐care testing that is easy to use and can provide results within minutes. These tests can help bridge the gap in low‐resource settings where laboratory infrastructure may be lacking [7]. Moreover, the development of multiplex assays capable of simultaneously detecting multiple viruses could enhance diagnostic accuracy and efficiency, enabling healthcare providers to make informed treatment decisions swiftly [4].

Additionally, recent advances in diagnostic technologies, such as CRISPR‐based detection platforms and isothermal amplification methods like LAMP, have not yet been discussed. CRISPR‐Cas‐based assays leveraging Cas12 and Cas13 collateral cleavage offer high sensitivity and specificity (attomolar detection limits), with rapid turnaround ( ~ 30 min) and minimal equipment requirements, making them well‐suited for deployment in resource‐limited environments. For instance, Zhao et al. [11] developed a one‐step LAMP–CRISPR/Cas12b assay for Monkeypox virus (MPXV) detection, achieving a limit of detection of 6.5 copies per reaction within 40 min and demonstrated 100% sensitivity and specificity in 113 clinical samples. Similarly, Zhao et al. [12] combined recombinase‐aided amplification (RAA) with CRISPR/Cas12a to detect MPXV at 10¹ copies/µL, with on‐site lateral‐flow strip readout. Isothermal amplification techniques such as LAMP and RPA also support multiplex detection and rapid turnaround under simple heating conditions. Integrating these methods with CRISPR‐Cas detection enhances specificity and makes multiplex point‐of‐care molecular diagnostics increasingly accessible, enabling better patient management, reduced complications, and faster outbreak control.

#### 3.1.5 Treatment Options for Mosquito‐Borne Viruses (MBVs)

##### 3.1.5.1 Current Treatments

The treatment options for MBVs primarily focus on symptomatic relief, as there are currently no specific antiviral therapies approved for most MBVs. For dengue virus, management typically includes the use of analgesics, such as acetaminophen (paracetamol), to alleviate pain and reduce fever. Non‐steroidal anti‐inflammatory drugs (NSAIDs) like ibuprofen and aspirin are generally avoided due to their potential to increase bleeding risk, especially in severe cases [7]. Hydration is also crucial, as maintaining fluid balance helps prevent complications, such as dengue shock syndrome. Patients are encouraged to drink plenty of fluids, and intravenous (IV) fluids may be administered in more severe cases to ensure adequate hydration [2].

For Zika and West Nile viruses, treatment is similarly supportive, focusing on managing symptoms, such as fever and pain. Rest, hydration, and the use of analgesics are recommended, while specific antiviral treatments remain unavailable [3]. Given the absence of targeted therapies for these viruses, managing symptoms and monitoring for complications are essential components of patient care.

##### 3.1.5.2 Antiviral Research

Ongoing research into antiviral therapies for MBVs is critical, as there is a pressing need for effective treatments. Various approaches are being explored, including the development of small‐molecule inhibitors, monoclonal antibodies, and vaccine candidates that could also provide therapeutic benefits. For instance, researchers are investigating compounds that inhibit viral entry or replication, aiming to reduce viral loads and improve clinical outcomes [8]. The success of such antiviral therapies would be a significant advancement, especially for severe cases of dengue or West Nile virus infections, which currently have limited treatment options. Additionally, the urgent need for effective treatments has been underscored by the increasing incidence of outbreaks globally, highlighting the necessity for continued investment in antiviral research.

##### 3.1.5.3 Public Health Interventions

In addition to medical treatments, public health interventions play a vital role in managing MBVs. Vector control strategies are essential for reducing transmission risks, as they aim to minimize mosquito populations and their breeding sites. Common strategies include insecticide spraying, larvicide, and community initiatives to eliminate standing water where mosquitoes breed [7].

Furthermore, the implementation of community health education programs is crucial for raising awareness about preventive measures, such as the use of mosquito repellents, bed nets, and environmental management to reduce mosquito habitats. Engaging communities in these efforts fosters a collaborative approach to disease prevention and empowers individuals to take proactive measures to protect themselves and their families from MBVs.

However, modern surveillance and predictive tools are still underutilized. Geographic Information System (GIS) based platforms integrated with machine learning and AI are enabling real‐time mapping of mosquito hotspots and environmental risk factors. These systems harness climatic, land‐use, and entomological data to visually display transmission risk zones and guide targeted interventions. Advanced predictive modeling using AI, such as Random Forest, support vector machines, LSTM neural networks, and graph neural networks, can forecast mosquito abundance or disease outbreaks weeks to months ahead, based on rainfall, temperature, and satellite imagery [13, 14]. Such tools enable precision public health responses by optimizing resource allocation and implementing timely vector control before outbreaks escalate.

#### 3.1.6 Research Gaps for Mosquito‐Borne Viruses (MBVs)

##### 3.1.6.1 Diagnostic Tools

One of the most pressing research gaps in the field of MBVs is the need for innovative diagnostic tools. Current diagnostic methods, while effective in many cases, often lack the rapidity and accessibility required for effective disease management, especially in low‐resource settings. There is a critical need for the development of point‐of‐care tests that can deliver accurate results quickly and without the need for complex laboratory infrastructure. Such tests would significantly enhance early diagnosis and timely treatment, particularly in areas where outbreaks occur. Additionally, multiplex assays capable of detecting multiple MBVs simultaneously would improve diagnostic accuracy and efficiency, addressing challenges posed by co‐infections and allowing for better public health responses [4]. Enhanced diagnostics would ultimately support better clinical decision‐making and contribute to more effective outbreak control strategies.

##### 3.1.6.2 Vaccine Development (Reviewer 4 Comment 2)

Vaccine development represents another significant area of need, particularly for MBVs, such as dengue, Zika, and West Nile viruses. While some vaccines exist, such as the dengue vaccine Dengvaxia, their efficacy varies by serotype and prior exposure, highlighting challenges in creating a universally effective vaccine [7]. There is an urgent need for innovative vaccine strategies that can provide broad protection across different serotypes and viruses. Current research efforts are exploring various platforms, including live attenuated, inactivated, and mRNA vaccines, but these initiatives face obstacles, such as safety concerns, the complexity of inducing long‐lasting immunity, and the need for rigorous testing in diverse populations [8].

However, next‐generation vaccine platforms have not been sufficiently emphasized. Recent advances in mRNA vaccine technology leveraging lipid nanoparticle (LNP)‐encapsulated mRNA encoding viral antigens have demonstrated robust immunogenicity and protective efficacy in preclinical models of dengue and Zika viruses [15, 16]. For example, nucleoside‐modified mRNA encoding Zika virus prM–E proteins delivered via LNPs elicited potent neutralizing antibody responses and protected mice from lethal challenge [17]. A similar approach for dengue virus has shown promise in generating tetravalent immune responses while avoiding antibody‐dependent enhancement [18]. Viral‐vectored platforms, such as adenovirus‐based and recombinant vesicular stomatitis virus (rVSV) vectors, are also being developed to target Zika and West Nile viruses, with demonstrated induction of both humoral and cellular immunity in animal models [19, 20]. These vector systems may enable single‐dose, thermostable vaccines suitable for use in low‐resource settings. Incorporating these next‐generation approaches could significantly accelerate MBV vaccine development, enhance cross‐serotype protection, and improve outbreak preparedness.

##### 3.1.6.3 Longitudinal Studies

The necessity for long‐term, longitudinal studies cannot be overstated, as these studies are essential for understanding the dynamics of MBV transmission, disease progression, and population‐level immunity. Current knowledge is often limited by short‐term studies that fail to capture the full spectrum of disease impact and vector behavior over time. Longitudinal research can provide valuable insights into the epidemiological trends of MBVs, the factors influencing outbreaks, and the effectiveness of interventions over extended periods [2]. Furthermore, such studies can inform public health strategies by identifying at‐risk populations and assessing the long‐term effects of infections, contributing to improved preparedness and response to future outbreaks. Enhanced understanding through longitudinal studies would ultimately support evidence‐based public health policies and resource allocation.

## 4 Discussion

This review provides an updated overview of MBVs, analyzing their geographic distribution, clinical impact, diagnostic challenges, and treatment options while identifying critical research gaps [5, 21]. Over the past several decades, the global incidence of MBVs has increased dramatically, posing growing challenges to public health. Dengue virus alone is responsible for approximately 390 million infections annually, and in 2024, the global dengue burden surged to over 14 million reported cases, with over 10,000 deaths. This rise is largely attributed to climate change, specifically El Niño, which has affected temperature and precipitation patterns across many parts of the world, enabling mosquito vectors like *Aedes aegypti* to thrive in previously unsuitable regions.

### 4.1 Geographic Distribution

The geographic distribution of MBVs has expanded far beyond the traditional tropical regions, now reaching temperate areas. This shift can be attributed to a combination of climate change and urbanization. Warmer temperatures and altered precipitation patterns have facilitated the spread of mosquito vectors, such as *Aedes aegypti* and *Culex quinquefasciatus*, extending the range of viruses like Zika, West Nile, dengue, Chikungunya, and yellow fever to higher latitudes and altitudes. A prime example of this expansion is Chikungunya virus, which was historically confined to Africa and Asia but has seen widespread outbreaks in regions such as the Caribbean, Latin America, and parts of Europe. Similarly, yellow fever, once largely confined to sub‐Saharan Africa and South America, remains a significant concern, with over 200,000 cases reported in 2024. El Niño conditions have exacerbated the situation by creating favorable breeding grounds for mosquitoes, contributing to increased transmission rates.

### 4.2 Climate Change and Predictions for MBV Spread

Several studies have explored how climate change affects the spread of MBVs, with rising temperatures and altered rainfall patterns expanding mosquito habitats and seasonal activity. Predictions indicate that, if climate trends continue, dengue, Zika, and West Nile virus could spread to new regions, including Southern Europe and parts of North America. Dengue, for example, could see an increase in transmission in temperate regions of Southern Europe and North America due to warmer winters and higher humidity [5]. Similarly, Zika outbreaks are expected to become more frequent in South America, Southeast Asia, and other tropical regions where *Aedes* mosquitoes thrive [2]. Additionally, West Nile virus is predicted to become more prevalent in regions where mosquito populations are expanding, particularly in Europe and parts of North America. Furthermore, integrating geographic information systems (GIS)‐based surveillance tools and artificial intelligence (AI)‐driven predictive models can significantly enhance monitoring and forecasting capabilities. Recent studies demonstrate how machine learning integrated with real‐time GIS platforms can anticipate mosquito abundance and dengue outbreaks with high accuracy [13, 14]. Predictive modeling using AI, such as neural networks and LSTM frameworks, has been shown to forecast outbreaks up to 2 months in advance [22], providing critical lead time for public health interventions.

### 4.3 Clinical Manifestations

The clinical manifestations of MBVs vary significantly depending on the virus. Dengue typically presents with high fever, severe headache, retro‐orbital pain, muscle and joint pain, rash, and mild bleeding. In severe cases, dengue can lead to dengue hemorrhagic fever and dengue shock syndrome, both of which require immediate medical intervention [23]. Zika virus infections are often asymptomatic or cause only mild illness, including fever, rash, and conjunctivitis. However, complications like Guillain‐Barré syndrome and birth defects in infants, such as microcephaly, can result from maternal infection during pregnancy [2]. Chikungunya typically causes high fever, severe joint pain (especially in the hands and feet), rash, and muscle pain. Joint pain can persist for weeks to months in some individuals, leading to long‐term disability and impaired quality of life (CDC, 2024). Similarly, yellow fever symptoms begin with fever, chills, headache, and muscle aches, but in severe cases, it progresses to jaundice, bleeding, and organ failure, which can result in death [24]. The mortality rate for severe yellow fever can be as high as 50%, particularly in areas with poor healthcare infrastructure and low vaccination coverage.

### 4.4 Diagnostic Challenges

Despite advances in diagnostics, the detection of MBVs remains challenging. PCR is a highly sensitive and specific method for detecting viral genetic material during the acute phase of infection. PCR is particularly useful for viruses such as Zika and West Nile, where timely diagnosis is critical for patient management [25]. However, PCR requires specialized laboratory equipment and skilled personnel, making it less accessible in resource‐limited settings. On the other hand, serological tests, such as ELISA, can detect antibodies like IgM and IgG, indicating past infections or immune responses. While ELISA tests are widely available, they are prone to cross‐reactivity between dengue and Zika, leading to false positives or false negatives [26]. There is an urgent need for the development of rapid, point‐of‐care diagnostics that are both affordable and effective, particularly in outbreak settings where quick diagnosis is critical. Notably, emerging diagnostic technologies such as CRISPR‐based detection and isothermal amplification methods like loop‐mediated isothermal amplification (LAMP) were not discussed in earlier reviews. These techniques offer promising rapid, sensitive, and field‐deployable diagnostic alternatives, especially useful in resource‐limited settings.

### 4.5 Treatment and Vaccine Development

Currently, treatment for MBVs is primarily supportive, focusing on symptomatic relief through the use of pain relievers and fluid management. There are no specific antiviral therapies for most MBVs, which represents a major gap in disease management. However, there has been progress in the development of vaccines. The dengue vaccine Dengvaxia has shown promise in preventing dengue in certain populations, though its use is controversial in seronegative individuals due to concerns about worsening disease severity in subsequent infections [27]. The yellow fever vaccine has been highly successful in preventing outbreaks, but coverage is still inadequate in some high‐risk regions, contributing to ongoing outbreaks in parts of sub‐Saharan Africa and South America. There are no licensed vaccines for Chikungunya or Zika, though research is ongoing to develop safe and effective vaccines for both viruses [28]. Recent advances in next‐generation vaccine platforms such as nucleoside‐modified mRNA vaccines and viral vector vaccines have shown promising results for Zika, dengue, and Chikungunya viruses [15, 17]. These platforms offer benefits, including rapid development timelines, strong immunogenicity, and potential for multivalent formulations, yet were not detailed in previous discussions.

### 4.6 Research Gaps and Future Directions

This review highlights the substantial research gaps in vaccine development, diagnostic methods, and treatment options for Zika, Chikungunya, and West Nile virus. Additionally, there is a pressing need for longitudinal studies that explore the long‐term epidemiological impacts of climate change on MBV transmission. As climate conditions continue to evolve, predicting the spread of these viruses is critical to public health preparedness. Studies have indicated that dengue, Zika, and West Nile will continue to expand their reach into previously unaffected regions, potentially leading to more frequent and severe outbreaks [5]. Collaborative efforts across research, public health, and policy sectors will be essential to address these gaps and reduce the burden of MBVs on global health systems.

## 5 Conclusion

In conclusion, this review underscores the growing public health threat posed by MBVs, exacerbated by climate change and urbanization. Our findings highlight that dengue alone affects over 100 countries with an estimated 390 million infections annually, while recent yellow fever outbreaks have exceeded 200,000 cases in 2024, emphasizing the scale of this global issue. The expansion of MBVs into new regions necessitates improved surveillance, rapid diagnostic tools, and effective treatment options. Despite the availability of some diagnostic methods, challenges, such as limited sensitivity in RDTs and cross‐reactivity in serological assays, hamper accurate detection, particularly in low‐resource settings. The development of vaccines for viruses, such as Zika, Chikungunya, and West Nile, remains a top priority. Although some vaccine candidates show promise, including dengue's Dengvaxia, their limited efficacy across serotypes and safety concerns highlight the need for next‐generation solutions. By addressing the identified research gaps, such as the development of multiplex diagnostic assays and long‐term epidemiological studies, the global community can better prepare for and mitigate the impact of these mosquito‐borne diseases on public health worldwide.

## Author Contributions

**Donath Damian:** conceptualization, investigation, funding acquisition, writing – original draft, methodology, validation, visualization, writing – review and editing, software, formal analysis, project administration, data curation, supervision, resources.

## Funding

The author received no specific funding for this work.

## Disclosure

The lead author Donath Damian affirms that this manuscript is an honest, accurate, and transparent account of the study being reported; that no important aspects of the study have been omitted; and that any discrepancies from the study as planned (and, if relevant, registered) have been explained.

## Conflicts of Interest

The author declares no conflicts of interest.

## Data Availability

This review does not contain original data. All information and insights presented in this manuscript are derived from previously published studies and publicly available literature. A comprehensive list of the references used in this review is provided. Data supporting the conclusions drawn from these studies can be accessed through the respective journals and repositories cited in the reference section.
